# Burden of disease and economic evaluation of healthcare interventions: are we investigating what really matters?

**DOI:** 10.1186/1472-6963-11-75

**Published:** 2011-04-13

**Authors:** Ferrán Catalá-López, Anna García-Altés, Elena Álvarez-Martín, Ricard Gènova-Maleras, Consuelo Morant-Ginestar, Antoni Parada

**Affiliations:** 1 Centro Superior de Investigación en Salud Pública (CSISP), Valencia, Spain; 2 Fundación Instituto de Investigación en Servicios de Salud, Valencia, Spain; 3 Catalan Agency for Health Information, Assessment and Quality (CAHIAQ), Barcelona, Spain; 4 Department of Preventive Medicine and Public Health, Rey Juan Carlos University, Madrid, Spain; 5 Primary Care General Directorate, Regional Health Council, Madrid, Spain; 6 Department of Health Information Systems, Regional Health Council, Madrid, Spain

**Keywords:** Disability-adjusted life years, Cost of illness, Cost-benefit analysis, Health economics, Spain

## Abstract

**Background:**

The allocation of limited available healthcare resources demands an agreed rational allocation principle and the consequent priority setting. We assessed the association between economic evaluations of healthcare interventions published in Spain (1983-2008) and the disease burden in the population.

**Methods:**

Electronic databases (e.g., PubMed/MEDLINE, SCOPUS, ISI Web of Knowledge, CRD, IME, IBECS) and reports from health technology assessment agencies were systematically reviewed. For each article, multiple variables were recorded such as: year and journal of publication, type of study, health intervention targetted, perspective of analysis, type of costs and sources of information, first author's affiliation, explicit recommendations aimed at decision-making, and the main disease cause to which the intervention was addressed. The following disease burden measures were calculated: years of life lost (YLLs), years lived with disability (YLDs), disability-adjusted life years (DALYs), and mortality by cause. Correlation and linear regression models were fitted.

**Results:**

Four hundred and seventy-seven economic evaluations were identified. Cardiovascular diseases (15.7%), infectious diseases (15.3%), malignant neoplasms (13.2%), and neuropsychiatric diseases (9.6%) were the conditions most commonly addressed. Accidents and injuries, congenital anomalies, oral conditions, nutritional deficiencies and other neoplasms were the categories with a lowest number of studies (0.6% for each of them). For the main disease categories (n = 20), a correlation was seen with: mortality 0.67 (p = 0.001), DALYs 0.63 (p = 0.003), YLLs 0.54 (p = 0.014), and YLDs 0.51 (p = 0.018). By disease sub-categories (n = 51), the correlations were generally low and non statistically significant.

**Conclusions:**

Examining discrepancies between economic evaluations in particular diseases and the overall burden of disease helps shed light on whether there are potentially over- and under-investigated areas. The approach taken could help policy-makers understand whether resources for economic evaluation are being allocated by using summary measures of population health.

## Background

Economic evaluation using clinical, epidemiological and economic data allows for a comparative analysis of alternative actions in terms of costs and health outcomes. Nowadays, the explicit use of the information from these studies is presumed to be a valuable tool for decision-making. Its consideration is justified by the limited amount of resources and is ethically grounded because those resources used inefficiently involve a lost opportunity to address other population health needs.

In recent decades the number of economic evaluations has increased over time [1] for several reasons, including: raised healthcare costs, population aging, epidemiological transition to chronic diseases, as well as continuous development of technological innovation and the explicit need to ensure rational use of existing healthcare resources. Ideally, research priorities should be defined based on the population health needs and efficient investments in interventions with proven benefits in health outcomes. The efficiency level could be measured by economic evaluation studies, while the population health needs may be evaluated through health losses attributable to fatal and non-fatal outcomes of the diseases, injuries and associated risk factors, also called *burden of disease *[3,4].

A substantial number of economic evaluations would be thus expected, aimed at measuring the efficiency of interventions for diseases with a high epidemiologic and socioeconomic burden. Some analyses have investigated the allocation of resources for health research in the past [5-9]. In the same line, Neumann *et al*. [10] analyzed the relationship between the disease burden and economic evaluations performed in the U.S. and other Western countries. The study by Neumann *et al*. was the first to explore research priorities for a specific type of economic evaluation in healthcare: cost-utility analyses. However, none of the systematic reviews of the use of economic evaluation published have approached this issue in Southern European countries such as Spain [1,2,11,12].

In this context, we examined whether full-economic evaluations of healthcare interventions (e.g., evaluations where both costs and outcomes have been measured) are aimed at those conditions generating a higher burden of disease. We expect that the information obtained would contribute to the current debate on establishing health priorities of future health technology assessment (HTA) research agendas.

## Methods

This is an observational study that summarizes the main characteristics of economic evaluation studies published in Spain (1983-2008), updates the calculation of the Burden of Disease study in the Spanish population, and investigates the association between economic evaluation research and the burden of disease in the population.

### Systematic review of economic evaluation studies

The results from a previous review that examined economic evaluation studies published within the years 1983-1999 [1], updated with the studies published until 2008 [2] were analyzed. Briefly, a systematic review was performed in PubMed/MEDLINE, SCOPUS, ISI Web of Knowledge, Databases of the Centre for Reviews and Dissemination (CRD), as well as *Índice Médico Español *(IME) and *Índice Bibliográfico Español en Ciencias de la Salud *(IBECS). Medical subject headings (MeSH) descriptors were used distributed into two blocks: economic evaluation and geographical area. For the section of geographical area, the search was based on a previously validated approach [13] to minimise bias regarding the indexing of geographical items. The full list of terms used is shown in the additional file 1: "Search terms used in the bibliographic review". Furthermore, manual searches were made for reports from HTA agencies and publications in specialized Spanish journals partially included in the abovementioned databases.

#### Selection criteria

We included full-economic evaluations (e.g., cost minimization analysis [CMA], cost-effectiveness analysis [CEA], cost-utility analysis [CUA] or cost-benefit analysis [CBA]) that evaluated interventions performed in Spain aimed at specific disease conditions. Review studies, editorials, and abstracts of congresses were excluded. If an article was found repeated in several publications, that published earlier and/or in a journal with higher impact factor was included.

#### Classification parameters

For each study selected, we obtained the following information: year and journal of publication, type of study, health intervention targetted, perspective of analysis (in terms of which costs are considered e.g., society, healthcare system, hospital, others), type of costs (e.g., direct or indirect) and sources of information, affiliation of the first author, explicit recommendations aimed at decision-making, and the main cause of disease to which the intervention or health program was addressed. The main disease cause was defined according to the Global Burden of Disease (GBD) study classification [3,4]. The source of funding was also included. In this case, the studies reviewed were considered to be private when they mentioned funding by any profit-making private setting or if any of the authors was working in a private company. The non-systematic use of the economic evaluation terms by some papers led to assigning the type of study after carefully reading the article, paying special attention to the costs, effectiveness measures used and presentation of the research results (e.g., cost per life year gained or cost per quality-adjusted life year). The studies were reviewed independently by two investigators, downloading the information in a predesigned database. Any disagreements were resolved by discussion.

### Updating the calculation of the burden of disease

The Burden of Disease study in the Spanish population is based on previous analyses. Methods are described in further detail elsewhere [9,14]. We estimated the burden of disease using disability-adjusted life years (DALYs), a time-based summary measure of population health combining the years of life lost (YLLs) for early death and the years of life lost due to the time living with disability (YLDs) [3,15]. Mortality data for disease specific causes of death were also presented. The sources of information used were: the population corresponding to the estimation of the National Institute of Statistics [16], deaths by gender, age and cause for 2006 from the national mortality records [17] and estimations for the WHO Euro-A sub-region of the GBD study recently published [4].

### Statistical analysis

A descriptive analysis was performed using frequency and percentage counts. The possible association between economic evaluations and burden of disease measures was analyzed using parametric (Pearson's *r*) and non-parametric correlation analyses (Spearman's *ρ*). The Kolmogorov-Smirnov and Shapiro-Wilk tests were applied to assess the normality of the data. Dependence among variables was investigated with linear regression models. In this case, the economic evaluations were the dependent variable and the disease burden measures the independent variable. As the different measures did not follow a normal distribution, the data were log-transformed.

## Results

### Main characteristics of the economic evaluations studies

Sixteen out of 87 identified studies from the 1983-1999 review [1] were excluded for not meeting the defined criteria. Four hundred and six articles met the selection criteria for the period 2000-2008, and were added to the 71 studies previously identified, obtaining a total sample of 477 studies (see additional file 2: "Flow diagram of systematic review to identify eligible studies") [2]. Table 1 details the characteristics of the studies reviewed. The CEA prevailed as the most common technique (62.5% of the total) and the health system perspective was the most commonly used (42.1%). In 70.0% of the studies, the main intervention evaluated was treatments, and 17.4% approached preventive interventions (94.0% of them with medical judgement, e.g., vaccines). Furthermore, a large part of the studies did not specify the funding source; in those stating it (267 studies), the profit-making (76.8%) prevailed over the non-profit making nature (23.2%).

**Table 1:** Main characteristics of the economic evaluation studies.

Characteristics	n (%)
**Study type**	

Cost-effectiveness analysis (CEA)	298 (62.5)
Cost-minimization analysis (CMA)	78 (16.4)
Cost-utility analysis (CUA)	73 (15.3)
Cost-benefit analysis (CBA)	28 (5.9)

**Health research activities**	

**Prevention of disease and conditions, and promotion of well-being**	83 (17.4)
Medically oriented (e.g. chemoprevention, vaccines)	78 (16.4)
Education/behaviour	5 (1.0)

**Detection, screening and diagnosis**	53 (11.1)
Screening	48 (10.1)
Resources and procedures	5 (1.0)

**Treatments and therapeutic interventions**	334 (70.0)
Medically oriented (e.g. pharmaceuticals)	263 (55.1)
Devices and procedures	33 (6.9)
Surgery	28 (5.9)
Education/behaviour	10 (2.1)

**Rehabilitation**	7 (1.5)

**Methods**	

Decision analysis	162 (34.0)
Observational studies	111 (23.3)
Not explicit	88 (18.4)
Markov or other simulation models	74 (15.5)
Clinical trials	42 (8.8)

**Perspective adopted**	

Healthcare system	201 (42.1)
Non explicit	121 (25.4)
Hospital	93 (19.5)
Society	59 (12.4)
Others	3 (0.6)

**Costs**	

Direct	404 (84.7)
Direct and indirect	73 (15.3)

**Cost information**	

Explicit	359 (75.3)
Non explicit	118 (24.7)

**Funding source**	

Non explicit	210 (44.0)
For profit	205 (43.0)
Non for profit	62 (13.0)

**Affiliation of the first author**	

Hospital	243 (50.9)
Private (e.g. consulting, pharmaceutical industry)	99 (20.7)
University	56 (11.7)
Administration	55 (11.5)
Primary care	17 (3.6)
Non explicit	7 (1.5)

**Recommendations**	

Yes	392 (82.2)
No	85 (17.8)

Cardiovascular diseases (15.7%), infectious and parasitic diseases (15.3%), and malignant neoplasms (13.2%) were the disease categories (n = 20) most commonly studied. Accidents and injuries, congenital anomalies, oral conditions, nutritional deficiencies and other neoplasms were the categories with a lowest number of studies (0.6% from the total for each of them) (Table 2). The disease sub-categories (n = 51) most prevalent in the studies were lower respiratory infections (5.7%), ischemic heart disease (5.7%), hepatitis B and C (3.3%) and HIV-AIDS (3.1%) (Table 3).

**Table 2:** Summary of burden of disease measures and economic evaluations for 20 disease-specific categories.

	National Burden of Disease in Spain 2006				Economic evaluations
Disease categories*	**DALYs in thousands (%)**	**YLLs in thousands (%)**	**YLDs in thousands (%)**	**Mortality in thousands (%)**	**Studies n (%)**

Neuropsychiatric conditions	1,467.9 (29.2)	110.9 (5.2)	1.356.9 (46.8)	28.7 (7.7)	46 (9.6)
Malignant neoplasms	798.7 (15.9)	720.2 (33.9)	78.5 (2.7)	101.0 (27.2)	63 (13.2)
Cardiovascular diseases	651.0 (12.9)	517.9 (24.4)	133.1 (4.6)	124.8 (33.6)	75 (15.7)
Sense organ diseases	409.2 (8.1)	0.0 (0.0)	409.2 (14.1)	0.0 (0.0)	12 (2.5)
Respiratory diseases	343.8 (6.8)	123.7 (5.8)	220.1 (7.6)	31.7 (8.5)	22 (4.6)
Accidents and injuries	326.3 (6.5)	241.6 (11.4)	84.7 (2.9)	16.1 (4.3)	3 (0.6)
Musculoskeletal diseases	234.3 (4.6)	11.4 (0.5)	222.9 (7.7)	3.5 (0.9)	31 (6.5)
Digestive diseases	213.6 (4.3)	120.8 (5.7)	92.8 (3.2)	20.0 (5.4)	30 (6.3)
Infectious and parasitic diseases	113.9 (2.3)	66.9 (3.1)	47.0 (1.6)	7.4 (2.0)	73 (15.3)
Diabetes mellitus	98.7 (2.0)	39.2 (1.8)	59.4 (2.0)	10.0 (2.7)	26 (5.4)
Endocrine and blood disorders	60.2 (1.2)	19.4 (0.9)	40.8 (1.4)	2.8 (0.7)	19 (4.0)
Genitourinary diseases	52.6 (1.0)	33.0 (1.5)	19.6 (0.7)	9.7 (2.6)	14 (2.9)
Congenital anomalies	51.0 (1.0)	27.2 (1.3)	23.8 (0.8)	1.0 (0.3)	3 (0.6)
Perinatal conditions	49.0 (1.0)	33.1 (1.6)	15.9 (0.5)	1.0 (0.3)	4 (0.8)
Respiratory infections	36.7 (0.7)	31.6 (1.5)	5.1 (0.2)	8.8 (2.4)	28 (5.9)
Oral conditions	34.4 (0.7)	0.1 (0.0)	34.3 (1.2)	0.0 (0.0)	3 (0.6)
Nutritional deficiencies	27.7 (0.5)	1.1 (0.0)	26.6 (0.9)	0.3 (0.1)	3 (0.6)
Other neoplasms	21.4 (0.4)	21.4 (1.0)	0.0 (0.0)	3.5 (0.9)	3 (0.6)
Maternal conditions	19.1 (0.4)	0.4 (0.0)	18.7 (0.6)	0.0 (0.0)	12 (2.5)
Skin diseases	11.0 (0.2)	3.4 (0.2)	7.6 (0.3)	1.1 (0.3)	7 (1.5)

**Total**	**5,020.8 (100)**	**2,123.6 (100)**	**2,897.2 (100)**	**371.5 (100)**	**477 (100)**

*Disease categories are presented in decreasing order of DALYs estimates in Spain (2006).

DALYs: Disability adjusted life years; YLLs: Years of life lost; YLDs: Years lived with disability.

**Table 3:** Disease-specific subcategories studied in economic evaluations compared to burden of disease measures.

Disease subcategories	DALYs % of total	Studies* % of total (rank)	Disease subcategories	YLLs % of total	Studies* % of total (rank)	Disease subcategories	YLDs % of total	Studies* % of total (rank)	Disease subcategories	Mortality % of total	Studies* % of total (rank)
Unipolar depression	8.3	1.9 (10)	Ischemic heart disease	9.2	5.7 (2)	Unipolar depression	14.3	1.9 (10)	Ischemic heart disease	10.3	5.7 (2)
Alzheimer's and other dementias	6.7	0.4 (28)	Lung cancer	7.8	2.5 (7)	Alzheimer's and other dementias	9.6	0.4 (28)	Cerebrovascular disease	9.2	1.3 (14)
Ischemic heart disease	4.6	5.7 (2)	Cerebrovascular disease	6.1	1.3 (14)	Hearing loss, adult onset	6.8	0.2 (40)	Alzheimer's and other dementias	5.9	0.4 (28)
Hearing loss, adult onset	3.9	0.2 (40)	Colorectal cancer	4.0	1.3 (15)	Macular degeneration	4.4	0.6 (24)	Lung cancer	5.7	2.5 (7)
Cerebrovascular disease	3.7	1.3 (14)	Breast cancer	2.7	2.5 (8)	Osteoarthritis	4.3	1.9 (11)	COPD	4.5	2.7 (5)
Lung cancer	3.4	2.5 (7)	COPD	2.7	2.7 (5)	Migraine	2.7	0.8 (19)	Colorectal cancer	3.8	1.3 (15)
Macular degeneration	2.6	0.6 (24)	Alzheimer's and other dementias	2.6	0.4 (28)	Drug use disorders	2.3	0.2 (41)	Lower respiratory infections	2.4	5.7 (1)
Osteoarthritis	2.5	1.9 (11)	Cirrhosis	2.3	0.6 (25)	Asthma	2.2	1.0 (17)	Hypertensive heart disease	1.9	1.9 (12)
COPD	2.3	2.7 (5)	Lymphomas, myelomas	1.5	0.4 (29)	Schizophrenia	2.2	1.9 (13)	Breast cancer	1.8	2.5 (8)
Colorectal cancer	2.0	1.3 (15)	Liver cancer	1.5	0.2 (43)	COPD	2.0	2.7 (5)	Nephritis, nephrosis	1.8	1.3 (16)
Migraine	1.6	0.8 (19)	Lower respiratory infections	1.5	5.7 (1)	Cerebrovascular disease	1.9	1.3 (14)	Prostate cancer	1.6	0.4 (31)
Breast cancer	1.4	2.5 (8)	Brain cancer	1.4	0.2 (44)	Rheumatoid arthritis	1.4	1.0 (18)	Cirrhosis	1.3	0.6 (25)
Drug use disorders	1.4	0.2 (41)	HIV- AIDS	1.3	3.1 (4)	Ischemic heart disease	1.2	5.7 (2)	Bladder cancer	1.3	0.2 (51)
Asthma	1.3	1.0 (17)	Leukemia	1.3	0.8 (22)	Panic disorder	1.2	0.2 (50)	Liver cancer	1.3	0.2 (43)
Schizophrenia	1.3	1.9 (13)	Bladder cancer	1.2	0.2 (51)	Falls	1.0	0.2 (42)	Lymphomas, myelomas	1.2	0.4 (29)
Cirrhosis	1.2	0.6 (25)	Nephritis, nephrosis	1.1	1.3 (16)	Parkinson disease	0.7	0.4 (30)	Leukemia	0.9	0.8 (22)
Falls	0.9	0.2 (42)	Prostate cancer	1.0	0.4 (31)	Colorectal cancer	0.6	1.3 (15)	Brain cancer	0.7	0.2 (44)
Rheumatoid arthritis	0.8	1.0 (18)	Hypertensive heart disease	1.0	1.9 (12)	Glaucoma	0.6	0.6 (26)	Parkinson disease	0.7	0.4 (30)
HIV/AIDS	0.8	3.1 (4)	Ovary cancer	0.8	0.2 (45)	HIV- AIDS	0.5	3.1 (4)	Kidney cancer	0.6	0.4 (32)
Panic disorder	0.7	0.2 (50)	Falls	0.7	0.2 (42)	Breast cancer	0.4	2.5 (8)	Falls	0.5	0.2 (42)

*Data from 1983-2008. Disease subcategories are presented in decreasing order of burden of disease estimates in Spain 2006. DALY: Disability adjusted life years; YLL: Years of life lost; YLD: Years lived with disability; COPD: Chronic Obstructive Pulmonary Disease; HIV- AIDS: Human Immunodeficiency Virus - Acquired Immunodeficiency Syndrome. Other disease subcategories covered by economic evaluation research (% of total studies, [% of total DALYs]): Lower respiratory infections (5.7, [0.6]), Hepatitis B and C (3.3, [0.2]), Peptic ulcer (2.7, [0.1]), Childhood vaccinable diseases (2.1, [0.04]), Epilepsy (0.8, [0.3]), Multiple sclerosis (0.8, [0.2]), Leukaemia (0.8, [0.6]), Cataracts (0.8, [0.1]), Laryngeal cancer (0.6, [0.3]), Benign prostatic hypertrophy (0.4, [0.2]), Melanoma (0.4, [0.2]), Cervical cancer (0.4, [0.2]), Appendicitis (0.4, [0.02]), Meningitis (0.4, [0.1]), Fires - unintentional injuries (0.4, [0.1]), Tuberculosis (0.4, [0.1]), Pancreatitis (0.2, [0.2]), Rheumatic heart disease (0.2, [0.2]) and Otitis media (0.2, [0.06]).

### Disease burden measures

Neuropsychiatric conditions were the main cause of DALYs accounting for 29.2%. Malignant neoplasms ranked second followed by cardiovascular diseases, generating 15.9% and 12.9%, respectively (Table 2). The subcategories selected (Table 3) included most remarkably the weight of DALYs in unipolar depression (8.3%), Alzheimer and other dementias (6.7%), ischemic heart disease (4.6%), age-related hearing loss (3.9%), cerebrovascular diseases (3.7%) and lung cancer (3.4%). Tables 2 and 3 show how the assessment of the impact of the various diseases in population health can change upon considering the effect of mortality alone or adding disability or poor health.

### Association between economic evaluation research and disease burden

**Figure 1:**
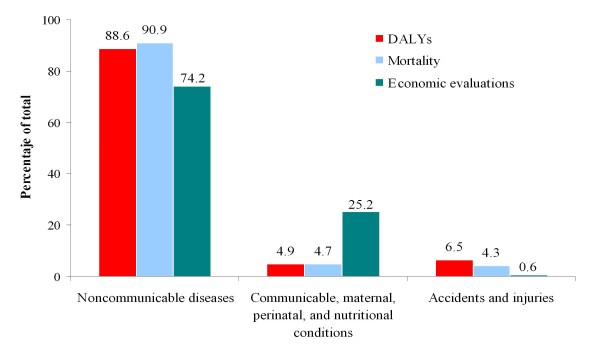
**Relationship between burden of disease measures and economic evaluation of healthcare interventions for the 3 broad disease groups of the Global Burden of Disease study**.

The interventions evaluated were aimed mainly at the study of non-communicable diseases (74.2% of the total) and this group in turn generates the highest mortality (90.0% deaths) and burden of disease (88.6% DALYs). There is a mismatch between DALYs and the number of economic evaluations. For example, communicable, maternal, perinatal and nutritional conditions accounted for 4.9% of DALYs and 25.2% of the economic analyses, while 0.6% of the studies were aimed at the study of accidents and injuries despite accounting for 6.5% of DALYs (see Figure 1). Neuropsychiatric conditions accounted for 29.2% of DALYs vs 9.6% of the economic evaluations (Table 2). In particular, depression and dementias were in the first positions of DALYs rankings for disease specific sub-categories, while in terms of economic studies, they ranked 10 and 28, respectively (Table 3). Similarly, sense organ diseases, respiratory diseases, or some cardiovascular diseases (such as stroke) and malignant neoplasms (lung and colorectal cancers) are high in DALY rankings yet had a low number of economic evaluations conducted. In contrast, infectious diseases, respiratory infections, diabetes or digestive diseases are over-represented in terms of economic reports as compared to their disease burden.

**Figure 2:**
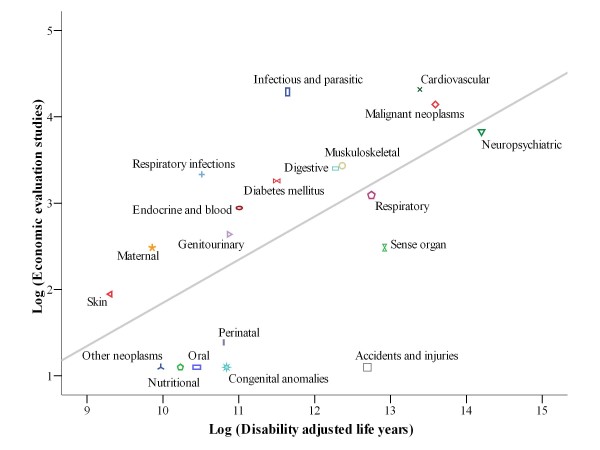
**Relationship between disability adjusted life years (DALYs) for 20 disease-categories and economic evaluation of healthcare interventions**. Note: The *x *and *y *axes are on logarithmic scales. R^2 ^= 0.358; Adjusted R^2 ^= 0.323. The line represents economic evaluation studies predicted on the basis of a simple linear regression model with disability adjusted life years (DALYs) as the explanatory variable.

The Spearman's correlation coefficients (*ρ*) between economic evaluations and burden measures were calculated. By disease categories (n = 20), a moderate correlation was seen between the various measures tested, the highest being for total mortality (*ρ *= 0.671; p < 0.001), followed by DALYs (*ρ *= 0.634; p = 0.003), YLLs (*ρ *= 0.540; p = 0.014) and YLDs (*ρ *= 0.508; p = 0.022). By sub-categories (n = 51), weak, non-statistically significant correlations were found (see data on Table 4). Data log-transformation allowed for calculating the Pearson's correlation coefficients (*r*), finding a moderate association by categories: 0.599 (p = 0.005) DALYs, 0.558 (p = 0.011) mortality, 0.473 (p = 0.035) YLLs and 0.399 (p = 0.091) YLDs. In the analysis of the main subcategories, there was only a statistically significant correlation in the case of DALYs, though it was still weak (*r *= 0.277; p = 0.049). For mortality, YLDs and YLLs, the correlation coefficients were not statistically significant: 0.206 (p = 0.169), 0.197 (p = 0.165), and 0.165 (p = 0.272). Figure 2 shows the actual and predicted values of the economic evaluation studies using the number of DALYs as predictive value in a linear regression model. The distance from the points corresponding to the categories to their projection in the line means the difference between the existing economic evaluation studies (observed values) and those that should be (expected values) if they reflected burden of disease.

**Table 4:** Spearman's correlation coefficients (*ρ*) between burden of disease measures and diseases covered by economic evaluations of healthcare interventions

Measure (year)	Correlation coefficient (n = 20)*	p-value	Correlation coefficient (n = 51)	p-value
Mortality (2006)	0.671	0.001	0.226	0.111

DALYs (2006)	0.634	0.003	0.267	0.058

YLLs (2006)	0.540	0.014	0.208	0.143

YLDs (2006)	0.508	0.022	0.226	0.111

* The results were statistically significant at a level 0.050 (bilateral).

DALYs: Disability adjusted life years; YLLs: Years of life lost; YLDs: Years lived with disability.

Units: Mortality (number of deaths per year), DALYs (years), YLLs (years) and YLDs (years).

## Discussion

The results of this analysis show that there is a mild to moderate association between economic evaluations and the burden of disease in the population. For some conditions, a lower number of reports have been seen considering the burden they generate, as in the case of accidents and injuries, some neuropsychiatric conditions, sense organ diseases, cerebrovascular diseases, some malignant neoplasms, or osteoarthritis. Furthermore, the mismatching of economic evaluations compared to the disease burden for infectious and parasitic diseases are to be noted. An explanation to this could be the high number of effective interventions available (e.g., antibiotics and vaccines) and that this has generated the need to perform economic evaluations, amongst others [18,19]. It could also be argued that the availability of interventions with a known efficiency profile could have generated some disinterest in performing new evaluations, thus leading to a lower number of reports, as could be the case for nutrition and physical activity interventions to prevent adverse cardiovascular outcomes. The lack of safety, efficacy and/or effectiveness data on some interventions, particularly in diseases such as Alzheimer's dementia or drugs abuse, could have decreased attention to the development of evaluations, and even, in case they had been performed, could have prevented disclosure of the findings given unfavorable results [20]. For some of the health conditions (such as accidents and injuries) where there have been a low number of economic studies conducted there might be a lesser number of researchers funded and/or interested in costing trauma prevention and rehabilitation measures than other diseases.

It seems that particularly economic evaluation research priorities are established by the interests and concerns of the pharmaceutical industry and the investigators or pressures from advocacy groups [21,22] which could explain the prevalence of evaluations in interventions to approach some diseases. With this regard, the results of our study are consistent with those of other previous studies that also evidenced disagreements between the allocation of research resources and some conditions with a substantial burden of disease [5,7,9,10]. In the seminal U.S.-based study, Gross *et al*. [5] examined the relationship between National Institutes of Health (NIH) funding and the burden of disease for 29 conditions. They found that research funding was more closely associated with DALYs than other burden measures such as mortality, prevalence, incidence or hospital days. Along the same line, Neumann *et al*. [10] conducted the first study to look at economic evaluation priorities. The authors found that cost-utility analyses were generally associated with burden measures for 50 specific conditions. Comparing our correlation coefficients to those obtained in the U.S. and other Western countries [10], the magnitude of the association between DALYs and economic evaluation studies was extremely poor: Spearman's *ρ *= 0.267 (p = 0.058) vs. *ρ *= 0.455 (p = 0.001). Our impression is that the health economics research we have examined is not oriented towards interventions to reduce the most burdensome diseases. We believe these findings do not necessarily imply that resources were allocated inefficiently, but rather raises important questions to keep in mind when funding future economic evaluations.

**Figure 3:**
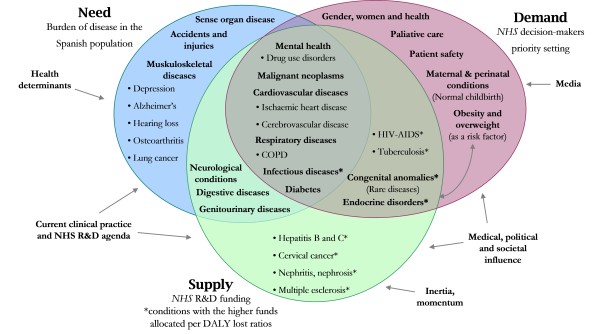
**Health priorities overlapping through different perspectives in Spain**. Note: Main disease categories (in boldface) and sub-categories (in drawings). The information source for NHS R&D funding classification was derived from: Catalá-López et al. 2009. Other sources: Burden of disease estimates (expressed as DALYs lost in 2006) proceeded from the present study. NHS priority-setting classification was established by the following actions: 1) Statement of the Minister of Health and Social Policy to inform on the general policy lines of the Department, at the Senate Commitee on Health. Madrid, June 18, 2009. Available from [in Spanish]: http://www.msc.es/gabinetePrensa/discursosInterv/archivos/180609122907.pdf [accessed June 25, 2009]. 2) Quality Plan for the National Health System. Madrid: Ministry of Health and Consumer Affairs, 2007. 3) Multisectorial Plan for HIV-AIDS: Spain 2008-2012. Madrid: Ministry of Health and Consumer Affairs, 2008. 4) Plan for the Prevention and Control of Tuberculosis in Spain. Madrid: Ministry of Health and Consumer Affairs, 2008. 5) The NAOS Strategy: a Strategy for Nutrition, Physical Activity, Obesity & Overweight Prevention. Madrid: Safety Food Agency - Ministry of Health and Consumer Affairs; 2005. 6) The Spanish National Drugs Strategy 2009-2016. Ruling of 2 February 2009, of the Government Commission for the National Abuse Drug Plan, publishing the Agreement of the Cabinet that approves the National Abuse Drug Strategy 2009-2016. Official State Journal (BOE) number 38 of February 13th 2009.

On the other hand, we further contextualized our results within the National Health System. Figure 3 shows the overlapping of health priorities distinguishing between: 1) Population health needs, influenced by issues such as health determinants, current research and development (R&D) agenda or the clinical practice in health services, 2) NHS decision-makers evaluation demand, that could be influenced by several scientific-professional societies, political groups and/or the community and by the mass media, and, finally, 3) Public resources relative to R&D in health, dependent on several pressures (e.g., political, medical and/or social), historic patterns, inertia or circumstancial problems when the allocation procedure occurs (e.g., recently the health crisis for pandemic influenza). In contrast, this research has identified the lack of economic evaluations aimed at public health interventions, diagnostic techniques, or rehabilitation. The subject bias of the evaluations identified in the review towards therapeutic interventions (generally medicines) would be due to the easier methods required for analysis. The efficacy of the treatments has been extensively estimated under experimental conditions, mainly through randomized clinical trials. Second, the pharmaceutical industry has allocated a high amount of resources to R&D with new products and these have been marketed within the time window studied, also simultaneously with the development and progressive addition of economic evaluation methods in the National Healthcare System. Several papers have demonstrated some form of cost-effectiveness is now required for healthcare interventions to be covered by many health administrations [23-25]. Then, it appears to be normal that insurers intend to evaluate the economic profitability of the new technologies and that private companies are promoting the efficiency of their products to enhance market access, pricing and reimbursement activities. The latter leads us to think about it from a public health standpoint, as private profit-making initiative investments do not necessarily intend to satisfy community preferences (or its health needs), significant health gains could be obtained from the resources available if health authorities prioritized the assessment of efficiency, preferably of interventions that have been extensively studied for a long time and that are aimed at identifying health problems that affect large population groups (or significant subpopulations) [26]. It is noteworthy to mention the fact that complementary approaches to prioritization exist in an effort to overcome the inadequacies when a criterion is used exclusively. In addition to the burden of disease and efficiency approaches, criteria of social justice, social solidarity, and equity amongst special patient groups (e.g., rare diseases, children, elderly people) may be conciliated in determining health priorities [26,27]. In the European Union, for exemple, the legislation for orphan medicinal products (Regulation EC No 141/2000) and for paediatrics (Regulation EC No 1901/2006) have succesfully included key elements to protect the interests of these patient groups.

Some limitations can be highlighted in our study. First, there is a lot of heterogeneity regarding the quality of the economic evaluations identified in terms of the type of intervention, targetted disease, methods, data sources, and costs included. However, for the purpose of analysis it has been considered that all studies included are equally significant, which may not always be true. Second, as in any review study, one could not rule out the potential non-identification of some studies or disagreements between the criteria of two reviewers. To minimize this bias we used the largest search possible, pre-defined inclusion criteria, and discussion of disagreements between the investigators. Furthermore, publication bias may occur, as noted above. There may be an even more important concern than bias, namely, screening a *priori *that may have been performed by the companies or producers of these analyses, which would make that economic evaluations would have been only funded in cases where a positive result was expected. These biases could have been approached only increasing economic evaluation funding from other sources, mainly public ones [28]. Third, we recognize there exists some arbitrary nature involved in clasifying economic evaluation studies to specific disease subcategories and other researchers may have classified them in a different way.

Finally, besides the DALYs, other alternative summary measures of population health have been proposed including quality-adjusted life years (QALYs) or healthy life years (HeaLY). The benefits and challenges of these measures have been examined [29,30]. As the DALYs has been the most widely-used measure in priority setting for global health, we focused on it in this study. Some of the limitations of the DALY approach refer to the parameters used for the their calculation, such as social preferences to establish the disability weights, discounting and age weighting. Furthermore, the methodology we used did not allowed to include co-morbities in the DALYs calculation, despite its importance in terms of quality of life, healthcare ressource use, costs and mortality [31-33]. Despite this fact, we think that DALYs are a measure of the most relevant health outcomes than theoretically more objective measures, such as deaths averted or life years gained (not adjusted). In future investigations, it would be interesting to perform an approach in line with the Australian burden of disease study [34], where for the first time DALYs were calculated considering co-morbidity of non-fatal conditions in the elderly age, some mental disorders, congenital anomalies and accidents, and the burden attributable to the main risk factors.

## Conclusions

The current need for measuring health losses in the population is unquestionable considering both the fatal and the disabling consequences of diseases to obtain a more complete information on the health problems. With this regard, estimating DALYs means an approach in the identification of immediate needs. Examining discrepancies between the numbers of economic evaluations in particular diseases and the overall burden of disease helps shed light on whether there are potentially over- and under-investigated areas. In terms of economic evaluative studies, this analysis shows that some research areas require greater attention by researchers and policy makers, specifically neuropsychiatric conditions, accidents and injuries, sense organ diseases, respiratory diseases, or some cardiovascular diseases (such as stroke) and malignant neoplasms (lung and colorectal cancers). Regulatory authorities, HTA agencies, universities or other public-private organizations could lead the change, dedicating more research lines to prevention, diagnosis and rehabilitation, diversifying interventions to conditions generating in the population a significant burden without neglecting other socially priority conditions (as in the case of rare diseases or paediatric populations).

Establishing health priorities is a complex process where multiple circumstances interfere (e.g. such as political decisions, the economic situation, etc.). Despite being one of the key points in the development of any health policy, many times making decisions about the health problems to be prioritized is accomplished unclearly and for reasons not always reasoned adequately. With this regard, the reduction of the burden of disease is an explicit criterion that, in combination with others such as efficiency (cost-effectiveness) and social equity, can allow for issuing recommendations to guide the debates about setting research priorities and, therefore, improving population health.

## Competing interests

The authors declare that they have no competing interests.

## Authors' contributions

All authors contributed to the development of the study, collected the data, interpreted the results, revised and commented the manuscript for important intellectual content and approved the final version.

## Pre-publication history

The pre-publication history for this paper can be accessed here:

http://www.biomedcentral.com/1472-6963/11/75/prepub

## Supplementary Material

Additional file 1 **Search terms used in bibliographic review**. The authors include the search terms used in the bibliographic review conducted in PubMed/MEDLINE, SCOPUS, CRD, ISI Web of Knowledge, IME and IBECS. Click here for file

Additional file 2 **Flow diagram of systematic review to identify eligible studies**. The flow diagram depicts the flow of information through the different phases of the systematic review of economic evaluations of healthcare interventions. Click here for file
