# Supplementary material for: Burden of disease and economic evaluation of healthcare interventions: are we investigating what really matters?
Source: BMC Health Serv Res. 2011 Apr 13;11:75. doi: 10.1186/1472-6963-11-75 (PMC3097252; doi:10.1186/1472-6963-11-75)
Supplement: Additional file 1 — Search terms used in bibliographic review. The authors include the search terms used in the bibliographic review conducted in PubMed/MEDLINE, SCOPUS, CRD, ISI Web of Knowledge, IME and IBECS. [file 1472-6963-11-75-S1.DOC]

**Additional file 1: Search terms used in bibliographic review**

**PubMed/MEDLINE**

Economic Evaluation section

Key words: Costs and cost analysis[mh] OR "cost-benefit analysis"[mh] OR "cost allocation"[mh] OR "cost control"[mh] OR "cost of illness"[mh] OR "cost savings"[mh] OR "cost sharing"[mh] OR "health expenditures"[mh] OR cost[ti] OR costs[ti] OR econom*[mh] OR econom*[ti] OR econom*[sh] OR saving*[ti] OR "fees and charges"[mh] OR reimburs*[ti] OR budget*[ti] OR expenditur*[ti] OR price[ti] or prices[ti] or pricing[ti] or pharmacoeconomic*[ti] OR finan*[ti]

Geographical ambit section

Key words: ("Spain"[MeSH Terms] OR spain[Text Word]) OR espagne[All Fields] OR espana[All Fields] OR (spain[ad] OR espagne[ad] OR espana[ad]) OR osasunbidea[ad] OR osakidetza[ad] OR insalud[ad] OR sergas[ad] OR (catalunya[ad] OR catalonia[ad] OR catalogne[ad] OR cataluna[ad] OR catala[ad] OR (barcelon[ad] OR barcelona[ad] OR barcelone[ad] OR barcelones[ad] OR barceloneta[ad]) OR tarragona[ad] OR lleida[ad] OR lerida[ad] OR girona[ad] OR gerona[ad] OR sabadell[ad] OR hospitalet[ad] OR l'hospitalet[ad]) OR ((valencia[ad] OR valenciana[ad] OR valenciano[ad]) OR (castello[ad] OR castellon[ad]) OR alacant[ad] OR (alicant[ad] OR alicante[ad]) OR (murcia[ad] OR murcian[ad] OR murciana[ad] OR murciano[ad])) OR ((andalucia[ad] OR andaluciajunta[ad] OR andalusia[ad] OR andalusian[ad] OR andaluz[ad] OR andaluza[ad]) OR (sevill[ad] OR sevilla[ad] OR seville[ad]) OR (granada[ad] OR granade[ad]) OR huelva[ad] OR almeria[ad] OR cadiz[ad] OR jaen[ad] OR malaga[ad] OR (cordoba[ad] NOT (argentina[ad]) OR (extremadura[ad] OR caceres[ad] OR badajoz[ad] OR madrid[ad]) OR (castilla[ad] OR salamanca[ad] OR zamora[ad] OR valladolid[ad] OR segovia[ad] OR soria[ad] OR palencia[ad] OR avila[ad] OR burgos[ad]) OR (leon[ad] NOT (france[ad] OR clermont[ad] OR rennes[ad] OR lyon[ad] OR USA[ad] OR (mexic[ad] OR mexica[ad] OR mexican[ad] OR mexicana[ad] OR mexicano[ad] OR mexicanos[ad] OR mexico[ad]))) OR (galicia[ad] OR gallego[ad] OR compostela[ad] OR vigo[ad] OR coruna[ad] OR ferrol[ad] OR orense[ad] OR ourense[ad] OR pontevedra[ad]) OR (oviedo[ad] OR gijon[ad] OR (asturia[ad] OR asturiano[ad] OR asturias[ad] OR asturias'[ad])) OR ((cantabria[ad] OR cantabrico[ad] OR cantabro[ad]) OR santander[ad]) OR (vasco[ad] OR euskadi[ad] OR basque[ad] OR bilbao[ad] OR bilbo[ad] OR (donosti[ad] OR donostia[ad]) OR vizcaya[ad] OR guipuzcoa[ad] OR gipuzkoa[ad] OR alava[ad] OR araba[ad] OR vitoria[ad]) OR ((navarra[ad] OR navarro[ad]) OR pamplona[ad] OR iruna[ad] OR irunea[ad]) OR ((aragon[ad] OR aragones[ad]) OR zaragoza[ad] OR teruel[ad] OR huesca[ad]) OR (mancha[ad] OR ciudad real[ad] OR albacete[ad] OR cuenca[ad]) OR (toledo[ad] NOT (ohio[ad] OR us[ad] OR usa[ad] OR OH[ad])) OR (guadalajara[ad] NOT (mexic[ad] OR mexica[ad] OR mexican[ad] OR mexicana[ad] OR mexicano[ad] OR mexicanos[ad] OR OR mexico[ad])) OR ((balear[ad] OR balearen[ad] OR baleares[ad] OR balearic[ad] OR balears[ad] OR balearse[ad]) OR mallorca[ad] OR menorca[ad] OR ibiza[ad] OR eivissa[ad]) OR (palmas[ad] OR lanzarote[ad] OR (canaria[ad] OR canarian[ad] OR canarias[ad] OR canario[ad]) OR tenerife[ad])

**SCOPUS**

((affil(spain or barcelona or madrid)) and ((title(cost or costs or econom*) or key(cost or costs or econom*)))) and (title(cost or costs or econom*)) and (limit-to(subjarea, "medi") or limit-to(subjarea, "phar") or limit-to(subjarea, "bioc") or limit-to(subjarea, "immu") or limit-to(subjarea, "nurs") or limit-to(subjarea, "heal") or limit-to(subjarea, "neur") or limit-to(subjarea, "psyc") or limit-to(subjarea, "dent") or limit-to(subjarea, "mult")) and (limit-to(pubyear, 2009) or limit-to(pubyear, 2008) or limit-to(pubyear, 2007) or limit-to(pubyear, 2006) or limit-to(pubyear, 2005) or limit-to(pubyear, 2004) or limit-to(pubyear, 2003) or limit-to(pubyear, 2002) or limit-to(pubyear, 2001) or limit-to(pubyear, 2000))

**ISI Web of Knowledge**

Title=(cost OR costs OR econom*) AND Address=(Spain)

Refined by: Subject Areas=( cardiac & cardiovascular systems or health care sciences & services or immunology or health policy & services or surgery or medicine, general & internal or pharmacology & pharmacy or public, environmental & occupational health or neurosciences or clinical neurology or gastroenterology & hepatology or medicine, research & experimental or psychiatry or rheumatology or genetics & heredity or hematology or oncology or transplantation or endocrinology & metabolism or urology & nephrology or respiratory system or infectious diseases )

**NHS-EED**

Key words: economic evaluation, cost-benefit, cost-effectiveness, cost-utility, Spain.

**HTA**

Key words: economic evaluation, cost-benefit, cost-effectiveness, cost-utility, Spain.

**Índice Médico Español (IME)**

Keywords: evaluación económica, coste* econom*, coste-efectividad, coste-beneficio, coste-utilidad.

**Índice Bibliográfico Español en Ciencias de la Salud (IBECS)**

Key words: evaluación económica, coste-beneficio, coste-efectividad, coste-utilidad.
