# Supplementary material for: Burden of disease and economic evaluation of healthcare interventions: are we investigating what really matters?
Source: BMC Health Serv Res. 2011 Apr 13;11:75. doi: 10.1186/1472-6963-11-75 (PMC3097252; doi:10.1186/1472-6963-11-75)
Supplement: Additional file 2 — Flow diagram of systematic review to identify eligible studies. The flow diagram depicts the flow of information through the different phases of the systematic review of economic evaluations of healthcare interventions. [file 1472-6963-11-75-S2.PPT]

## Slide 1
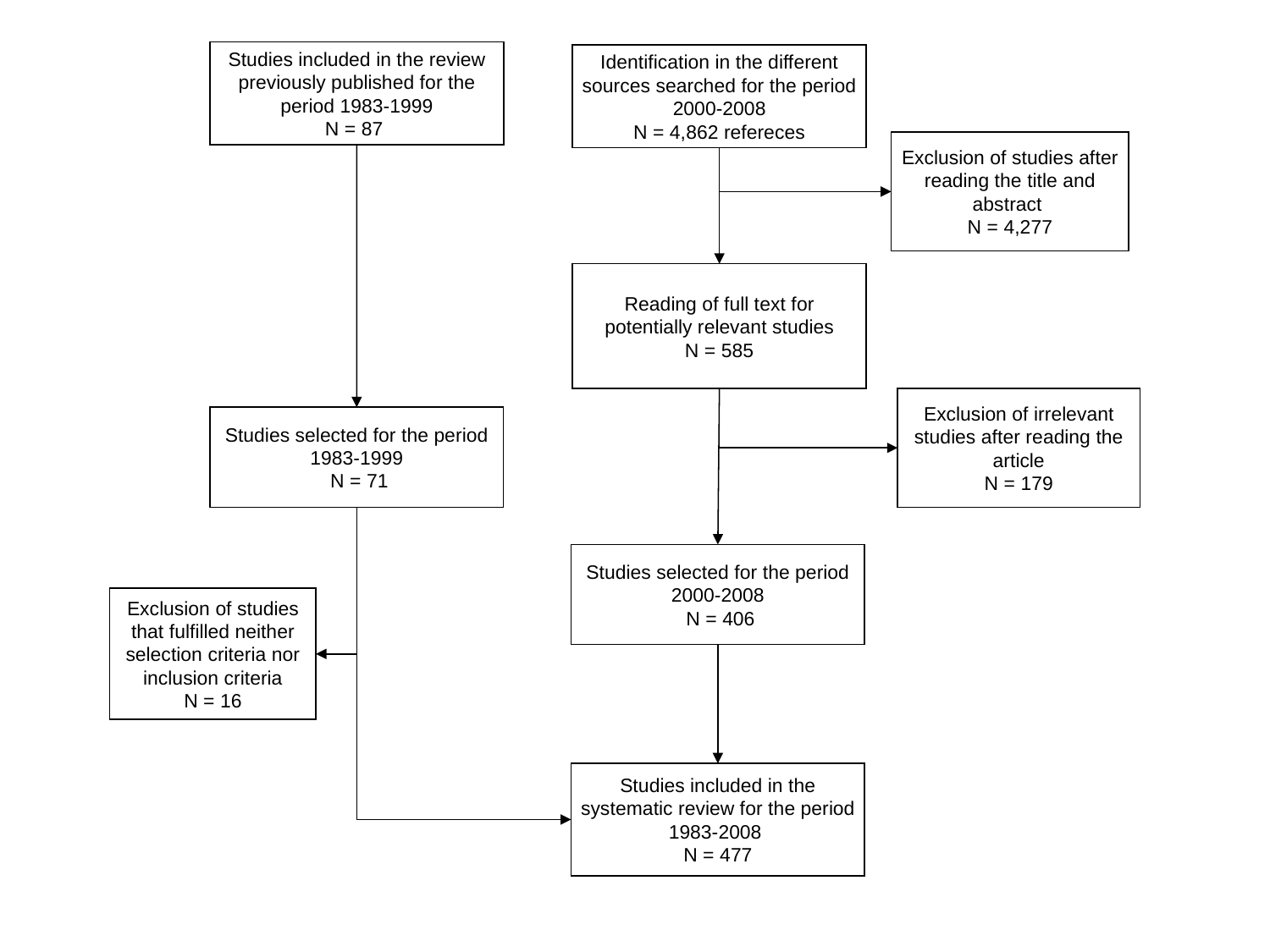

Studies included in the review previously published for the period 1983-1999
N = 87
Identification in the different sources searched for the period 2000-2008
N = 4,862 refereces
Exclusion of studies after reading the title and abstract
N = 4,277
Reading of full text for potentially relevant studies
N = 585
Exclusion of irrelevant studies after reading the article
N = 179
Studies selected for the period 1983-1999
 N = 71
Studies selected for the period 2000-2008
 N = 406
Exclusion of studies that fulfilled neither selection criteria nor inclusion criteria
N = 16
Studies included in the systematic review for the period 1983-2008
N = 477
